# Pollen-based reconstruction of vegetational and climatic change over the past ~30 ka at Shudu Lake in the Hengduan Mountains of Yunnan, southwestern China

**DOI:** 10.1371/journal.pone.0171967

**Published:** 2017-02-09

**Authors:** Yi-Feng Yao, Xiao-Yan Song, Alexandra H. Wortley, Yu-Fei Wang, Stephen Blackmore, Cheng-Sen Li

**Affiliations:** 1 State Key Laboratory of Systematic and Evolutionary Botany, Institute of Botany, Chinese Academy of Sciences, Xiangshan, Beijing, China; 2 Shanxi Agricultural University, Taigu, Shanxi, China; 3 Royal Botanic Garden Edinburgh, 20a Inverleith Row, Edinburgh, United Kingdom; Agharkar Research Institute, INDIA

## Abstract

The Hengduan Mountains, with a distinct altitudinal differentiation and strong vertical vegetation zonation, occupy an important position in southwestern China as a global hotspot of biodiversity. Pollen analysis of lake sediments sampled along an altitudinal gradient in this region helps us to understand how this vegetation zonation arose and how it has responded to climate change and human impacts through time. Here we present a ~30-ka pollen record and interpret it in terms of vegetational and climatic change from a 310 cm-long core from Shudu Lake, located in the Hengduan Mountains region. Our results suggest that from 30 to 22 cal. ka BP, the vegetation was dominated by steppe/grassland (comprising mainly *Artemisia*, Poaceae and Polygonaceae) and broad-leaved forest (primarily *Quercus*, *Betula* and *Castanopsis*) in the lake catchment, reflecting a relatively warm, wet climate early in this phase and slightly warmer, drier conditions late in the phase. The period between 22 and 13.9 cal. ka BP was marked by a large expansion of needle- and broad-leaved mixed forest (*Pinus*, *Abies* and *Quercus*) and a decline in the extent of steppe/grassland, indicating warming, drying climatic conditions followed by a cold, wet period. Between 13.9 and 3 cal. ka BP, steppe/grassland expanded and the area covered by needle- and broad-leaved mixed forest reduced, implying a fluctuating climate dominated by warm and humid conditions. After 3 cal. ka BP, the vegetation was characterized by an increase in needle-leaved forest and reduction in steppe/grassland, suggesting warming and drying climate. A synthesis of palynological investigations from this and other sites suggests that the vegetation succession patterns seen along an altitudinal gradient in northwestern Yunnan since the Late Pleistocene are comparable, but that each site has its own characteristics probably due to the influences of altitude, topography, microclimate and human impact.

## Introduction

The Hengduan Mountains lie to the eastern end of the Himalayan range, extending from western Sichuan and northern Yunnan to eastern Tibet in China and into northernmost Myanmar. This region is characterized by five main north-south oriented mountain ridges separated by four deep valleys with a distinct altitudinal differentiation ranging from 2000 m to 6000 m above sea level. This dramatic landscape was created during the Himalayan orogeny beginning in the Tertiary Period and continuing into the Quaternary Period [1–3].

The special natural and geographical characteristics of the area enable tropical weather systems from the south to penetrate northwards along the valleys, bringing a milder and wetter climate than occurs in less mountainous regions at similar latitudes [4]. Furthermore, the immense altitudinal range of the mountains creates a variety of different vegetation zones, superimposed on which is the influence of aspect, with the eastern and western flanks of the mountains providing different conditions for plant growth within a small area. Therefore, this region is very rich in floristic diversity, particularly in endemic species and genera. It was defined as the ‘Hengduan Mountains Hotspot’ by Boufford & Van Dyck [5], and now occupies a key position in southwestern China as a global hotspot of biodiversity.

Pollen analysis is recognized as a powerful tool for deciphering the regional responses of vegetation to climatic change and anthropogenic influences during the Quaternary [6–9]. Through pollen analysis of lake core sediments sampled along an altitudinal gradient in the Hengduan Mountains region, our objective is to develop a more detailed understanding of vegetational and climatic change in the Hengduan Mountains, through geological time, than can be obtained from any individual site.

One of the sites studied is Shudu Lake, currently, a nationally protected area and popular tourist destination in Shangri-la County. Due to its important geographical position in southwestern China, within the Hengduan Mountains Hotspot and the southwest monsoon-dominated region, it has been the focus of a diverse range of studies covering metal geochemistry [10], human activity [11], climate evolution [12], environmental change [13], lake ecosystem dynamics [14] and Asian palaeomonsoon variability [15, 16]. This paper presents a ~30-ka pollen sequence from Shudu Lake, and is the longest core, in terms of timescale, in a series of ongoing studies covering a range of altitudes within the Hengduan Mountains biodiversity hotspot. It aims to reconstruct the history of vegetational and climatic change over the past ~30 ka at Shudu Lake interpreted from palynological evidence. Furthermore, a synthesis of published palynological literature together with our own research work has been compiled to understand the vegetation succession patterns along an altitudinal gradient in northwestern Yunnan.

## Materials and methods

### Ethics statement

All necessary permits were obtained for the described field studies and were granted by the local government of Yunnan Province.

### Research site

**Fig 1:**
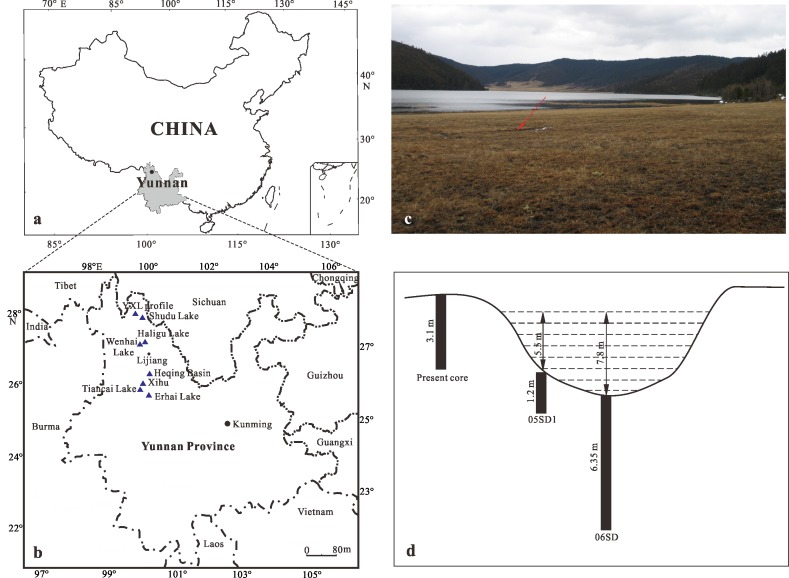
a. Location of Yunnan in China, b. Location of Shudu Lake and other studied sites in northwestern Yunnan, c. Panoramic view of Shudu Lake (red arrow represents position of core), d. Position of present and previous studied cores at Shudu Lake.

Shudu Lake (27°54′19″ N, 99°56′18″ E, altitude: 3620 m) is an open glacial lake, located ca. 35 km northeast of Shangri-la (Zhongdian) County in the Hengduan Mountains of Yunnan, southwestern China (Fig 1A and 1C). It is a high altitude, medium-sized lake situated on the southeastern edge of the Qinghai-Xizang (Tibetan) Plateau, within the Shangri-la Pudacuo National Park and the Three Parallel Rivers Scenic Area World Heritage Site [17, 18]. The lake is located about 600 m below the present tree line (within the alpine/subalpine ecotone) and catchment vegetation is highly sensitive to environmental and climatic changes [13].

The lake catchment covers an area of 14.2 km^2^ and a surface area of 1.7 km^2^. The diameter of the lake is 2.27 km with a maximum water depth of about 7.8 m. The catchment geology is diverse including quartzite, mica schist, limestone and sandstone [14, 15] and this is reflected in the high floristic diversity. The southwest monsoon from the Indian Ocean prevails in this region, thus the summers are warm and humid and the winters cool and dry. The mean annual temperature and precipitation are 5.7°C and 640 mm, respectively, as recorded by the Zhongdian Meteorological Station between 1958 and 2002 [12].

The study area is located within a vegetation domain characterized by alpine *Picea*-*Abies* forest, alpine scrub and meadow [19]. Based on our personal observations in the field, the local vegetation is dominated by the following families and genera: needle-leaved trees including *Picea*, *Abies* (e.g. *A*. *georgei* Orr), and *Tsuga*; broad-leaved trees and shrubs including *Quercus* (e.g. *Q*. *aquifolioides* Rehd. et Wils), *Betula*, *Salix* and Ericaceae; and upland herbs including Poaceae, Cyperaceae, Gentianaceae and Polygonaceae. In addition, a large number of mosses (e.g. *Sphagnum* sp.) and lichens (e.g. *Usnea longissima* Ach., *Lobaria pulmonaria* (L.) Hoffm.) are visible growing on the ground surface and trees in the lake catchment due to the abundant surface moisture and high relative humidity.

### Core and lithology

**Fig 2:**
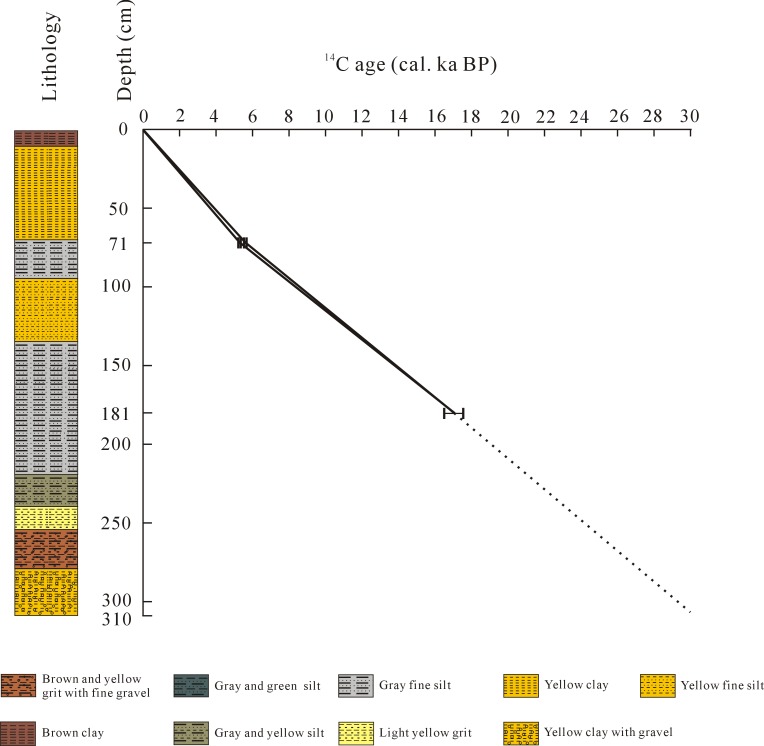
Lithology of the Shudu Lake core and age-depth curve showing rate of sedimentation.

A 310 cm-long core was obtained from a site near the bank of Shudu Lake (Fig 1C and 1D) in March 2005, using a Russian corer. The sediment core is mainly composed of silt, clay and grit. A detailed description of the lithology is as follows: 310–280 cm, yellow clay with gravel; 280–255 cm, brown and yellow grit with fine gravel; 255–240 cm, light yellow grit; 240–220 cm, gray and yellow silt; 220–135 cm, gray and green silt; 135–95 cm, fine yellow silt; 95–70 cm, gray and green silt; 70–10 cm, yellow clay; 10–0 cm, brown clay (Fig 2).

### Dating method

Three samples from the core, at 71 cm, 181 cm and 310 cm in depth, were extracted for accelerator mass spectrometry (AMS) radiocarbon dating, which was undertaken at the Scottish Universities Environmental Research Centre (SUERC) in Glasgow, Scotland, UK. ^14^C ages are quoted in conventional years before present (before AD 1950). Owing to the lack of suitable fragments of plant material, bulk samples from the core were used for dating. Age calibration was established using a calibration curve from Reimer *et al*. [20] by means of the calibration program OxCal v3.10 [21]. Date ranges are cited in calibrated years AD/BC at 95% probability, with end points rounded out to 10 years [22, 23].

### Pollen analysis

The core was sub-sampled at 10 cm intervals; in total thirty-one samples were taken from the core for pollen analysis. In addition, eight surface soil samples in close proximity to the core were collected for comparison with the preserved pollen assemblage. Thirty grams of each sample were processed by heavy liquid separation [24, 25] followed by acetolysis [26]. All samples were placed in 50% glycerine jelly and mounted on glass slides for light microscope observation. Pollen and spores were identified using modern pollen slides, palynological literature and monographs [27–29]. All samples yielded abundant, well-preserved palynomorphs. At least 300 pollen and spores were counted in each sample under a Leica DM 2500 light microscope. Pollen and spores were divided into four categories: trees and shrubs, herbs, pteridophytes and aquatic taxa. Pollen percentages and pollen diagram plotting were conducted with Tilia and Tilia Graph [30].

## Results

### Chronology

Due to a lack of humic acid, the sample from 310 cm failed to be dated. Consequently, two AMS dates were obtained for the core (Table 1) which, nevertheless, provided a relatively reliable chronology for interpreting the vegetation and climate history at Shudu Lake. An age-depth curve in cal. yr BP, reflecting the sedimentation pattern, was constructed for the core (Fig 2), and shows a similar sedimentation rate for the time intervals 17–5.4 cal. ka BP (ca. 0.10 mm/yr) and 5.4–0 cal. ka BP (ca. 0.13 mm/yr). The chronology at 40 cm (ca. 3 cal. ka BP) and 150 cm (ca. 13.9 cal. ka BP) depth was produced by linear interpolation between the dated depths shown on Fig 2. Given that the sedimentation rate below 181 cm was in agreement with that of the upper part, the dates at 230 cm and 310 cm were inferred to be ca. 22 cal. ka BP and ca. 30 cal. ka BP assuming a more or less steady accumulation of sediment in the study site (S1 Table).

**Table 1:** Results of radiocarbon dating.

Sample code	Depth (cm)	Material dated	δ^13^C‰ (VPDB)	^14^C yr BP	Cal. yr BP	Cal. yr AD/BC (2 sigma range)
SUERC-12764	71	Humic acid	–26.4	4740±35	5590–5440 5390–5320	3640–3490 BC 3440–3370 BC
SUERC-12765	181	Humic acid	–26.4	14240±45	17450–16550	15500–14600 BC

### Surface sample record

**Fig 3:**
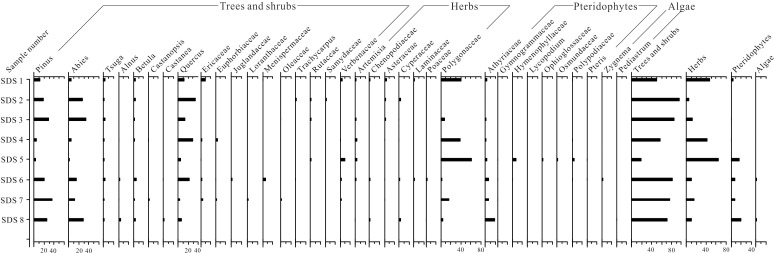
Pollen percentage diagram of major palynomorphs for Shudu Lake surface samples, Yunnan.

In total 47 different palynomorphs were identified from the eight surface soil samples, comprising 28 families and six genera of angiosperms, three genera of gymnosperms, seven families and one genus of pteridophytes, and two algal genera (Fig 3). The modern pollen assemblage is dominated by trees and shrubs, which provide between 50% and 94.9% of the pollen sum, with the exception of one sample (SDS 5: 19.6%). *Pinus*, *Abies* and *Quercus* pollen dominate in most samples with a maximum level of 35%. Herbaceous pollen reaches proportions of 42.3–63.7% in samples SDS 1, 4, 5, while remaining at relatively low percentages (5.1–16.2%) in the remainder. Polygonaceae pollen is predominant amongst the herbs (up to 60.9% in sample SDS 5). *Artemisia*, Asteraceae, Chenopodiaceae, Cyperaceae, Laminaceae and Poaceae pollen are present at low percentages (< 3% in most samples). Pteridophyte spores are represented by Athyriaceae (0–19%). In addition, Cyatheaceae, Gymnogrammaceae, Osmundaceae and *Pteris* were found in minute quantities (< 1%). Two types of algae, *Zygnema* (0–1.3%) and *Pediastrum* (0–0.7%), are recorded in samples SDS 6 and 8, respectively. This pollen assemblage is generally congruent with the local vegetation around the lake catchment, reflecting a needle-leaved forest (dominated by *Abies*) coupled with some broad-leaved components, e.g., *Quercus*, *Betula* and Ericaceae.

### Pollen diagram zonation and description

**Fig 4:**
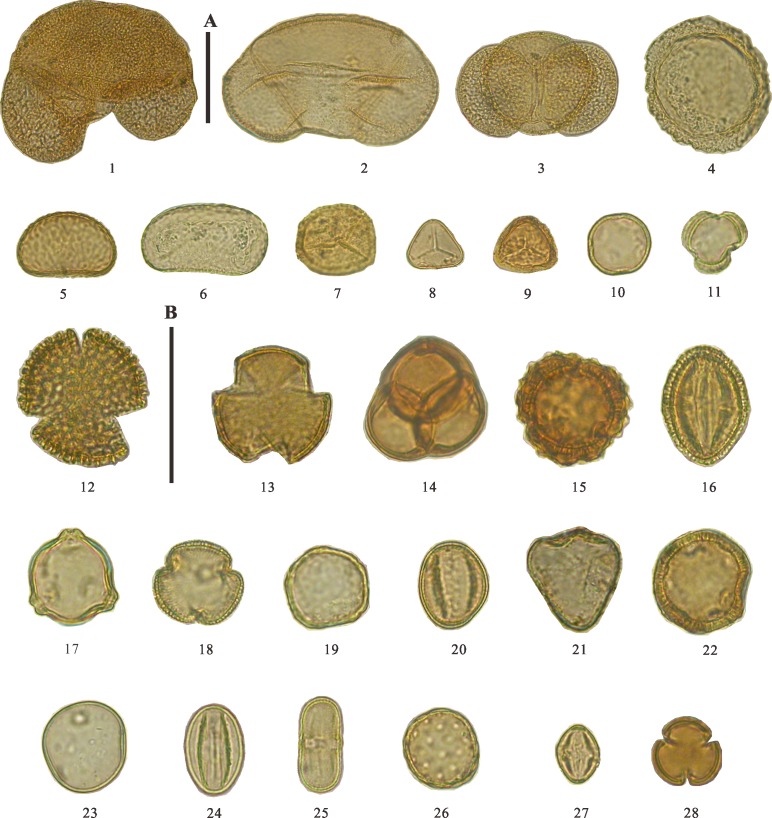
Examples of palynomorphs recovered from Shudu Lake core sediments 1. *Abies* L. 2. *Picea* Dietr. 3. *Pinus* L. 4. *Tsuga* Carr. 5. Polypodiaceae 6. Athyriaceae 7. Hymenophyllaceae 8. Gymnogrammaceae 9. *Pteris* L. 10. Plantaginaceae 11. *Artemisia* L. 12. Oleaceae 13. Rosaceae 14. Ericaceae 15. Asteraceae 16. Polygonaceae 17. *Betula* L. 18. Verbenaceae 19. *Ulmus* L. 20. *Quercus* L. 21. Cyperaceae 22. Amaranthaceae 23. Poaceae 24. *Salix* L. 25. Apiaceae 26. Chenopodiaceae 27. *Castanopsis* (D. Don) Spach. 28. Fabaceae Scale bar = 50 μm (scale bar A applies to 1–9, scale bar B applies to 10–28).

**Fig 5:**
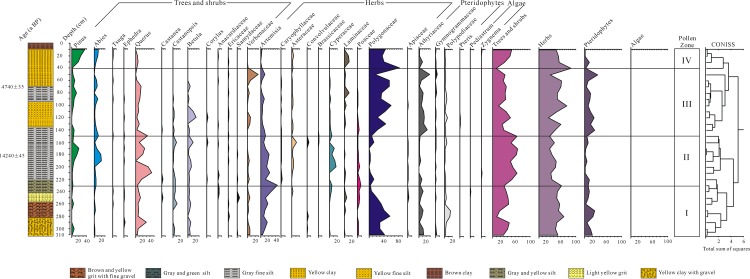
Pollen percentage diagram for major palynomorphs from Shudu Lake core, Yunnan.

The pollen and spores extracted from the Shudu Lake core samples show a high degree of taxonomic diversity. The palynoflora consists of 77 palynomorphs, which were assigned to 49 families and 10 genera of angiosperms, five genera of gymnosperms, nine families and two genera of pteridophytes, and two genera of algae. Selected palynomorphs are illustrated in Fig 4. The pollen diagram was divided into four distinct zones based on a cluster analysis using Tilia (with CONISS) (Fig 5). Each pollen zone is described below.

*Pollen Zone I (310–230 cm*, *30–22 cal*. *ka BP)*: This zone is characterized by the dominance of herbaceous pollen (45.5–67.9%), followed by trees and shrubs (10.7–44.7%), pteridophytes (2.5–27%) and aquatics (0–1.2%). Tree and shrub pollen is dominated by broad-leaved taxa such as *Quercus* (4.8–29.8%), *Castanopsis* (0–11.1%) and *Betula* (0–8.6%). Coniferous trees comprise mainly *Pinus* (0–6.5%), *Abies* (0–5.9%) and *Tsuga* (0–2.3%). Herb pollen is represented by Polygonaceae (2.9–57.1%) and *Artemisia* (4.8–44.1%), accompanied by Poaceae (0–8.8%), Cyperaceae (0–5.9%) and Laminaceae (0–5.4%). Six types of ferns were recorded, including Polypodiaceae (1.2–16.2%), Athyriaceae (1.2–13.3%), Loxogrammaceae (0–2.9%), Gymnogrammaceae (0–1.8%), Hymenophyllaceae (0–1.2%) and *Pteris* (0–0.6%). One alga, viz., *Pediastrum* was found in minute quantities (up to 1.2%).

*Pollen Zone II (230–150 cm*, *22–13*.*9 cal*. *ka BP)*: In this zone, tree and shrub pollen (47.9–67.3%) predominates rather than pollen of herbaceous plants. Compared with pollen zone I, herbaceous pollen and fern spores decline to 29.7–46.8% and 0.8–12.9%, respectively. Aquatics remain at low levels (0–0.6%). Coniferous trees such as *Pinus* (2.3–19%) and *Abies* (1.5–20.3%), and broad-leaved trees such as *Quercus* (1.4–44.6%), *Betula* (1.7–13.9%) and *Castanopsis* (0–9.1%) are the main contributors to the rise of tree and shrub pollen. A decrease in herbaceous pollen is attributed to a marked decline in Polygonaceae (0.8–13%) and a slight decrease in *Artemisia* (8.3–24.2%), while Cyperaceae (0–17.4%) and Asteraceae (0–11.7%) increase distinctly. Poaceae (0.5–6.1%) retains the same proportion as in pollen zone I. Athyriaceae (0–7.6%) and Polypodiaceae (0–6.1%) display a prominent decrease in this zone. Two algae, *Zygnema* and *Pediastrum*, occur sparsely.

*Pollen Zone III (150–40 cm*, *13*.*9–3 cal*. *ka BP)*: Herbaceous pollen dominates in this zone (26.7–87.1%), followed by trees and shrubs (9.5–48.1%), and pteridophytes (3.4–35.6%). No aquatics were found during this period. In contrast with pollen zone II, *Pinus* (0.8–12.4%), *Abies* (0–9.3%) and *Quercus* (0–14.6%) pollen decline, but *Betula* and Verbenaceae increase slightly, to peak values of 22.1% at 120 cm and 28% at 50 cm, respectively. Of the herb pollen, an abrupt rise in Polygonaceae is noted, reaching a maximum for the entire core of 82.8% at 40 cm. Laminaceae pollen increases to 12.9%. Pollen of *Artemisia*, Cyperaceae, Asteraceae and Poaceae decreases. Ferns are represented by a marked increase in Athyriaceae (1.4–29.5%). In addition, Polypodiaceae (1.5–7.6%), Gymnogrammaceae (0–3.8%) and *Pteris* (0–1.5%) also show a slight increase in proportion.

*Pollen Zone IV (40–0 cm*, *3 cal*. *ka BP–present)*: In this zone, herbaceous pollen (46.8–55.4%) and pteridophyte spores (1.5–13.1%) decline, but pollen of trees and shrubs increases (31.4–50.1%). The aquatics remain at low proportions (0–1.3%). Of the coniferous pollen types, *Pinus* shows a prominent rise (18.3–35.5%), with *Abies* pollen also displaying a slight increase (4.6–10.7%). The broad-leaved tree *Quercus* decreases to 1–6.9%, with *Betula* (0–0.6%) and Ericaceae (0–1.1%) occurring in minute quantities. Verbenaceae pollen disappears in this zone. Amongst the herbs, Polygonaceae always maintains a high level (36.3–43.3%), although it decreases compared with pollen zone III. Laminaceae (0–13.1%) shows a slight increase. *Artemisia* declines to 0–2.6%. Cyperaceae and Poaceae are absent in this zone. Fern spores decline, mainly attributed to a decrease in Athyriaceae, Gymnogrammaceae and Polypodiaceae. One alga, *Zygnema*, is documented at a low percentage (0–1.3%).

## Discussion and conclusions

### History of vegetational and climatic change over the past ~30 ka

**Fig 6:**
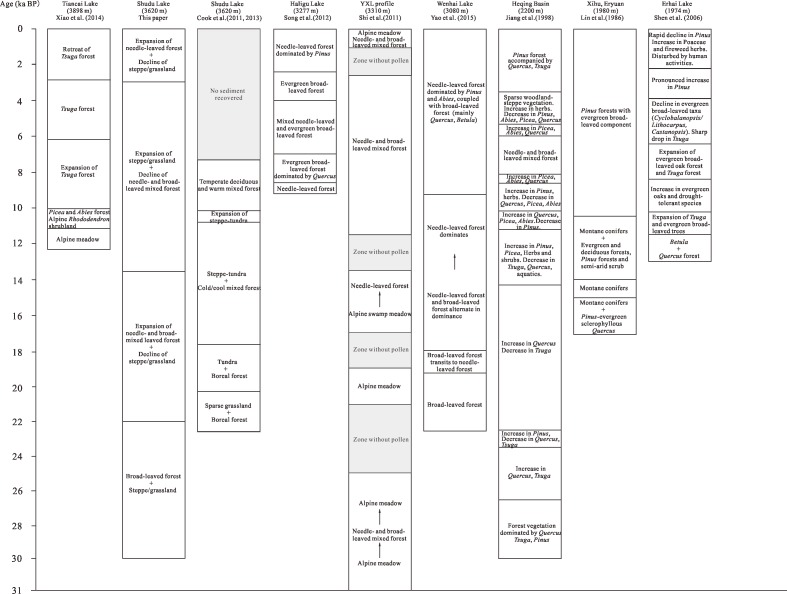
Regional comparison of vegetational succession in northwestern Yunnan.

The pollen sequence presented here, and compared with other studies in the same region, reveals the history of vegetation and climate change in the Shudu Lake catchment during the past ~30 ka (Fig 6). From 30 to 22 cal. ka BP (Pollen Zone I), the vegetation is inferred to have been dominated by steppe/grassland (mainly composed of *Artemisia*, Poaceae and Polygonaceae) around the lake, and broad-leaved forest (primarily *Quercus*, *Betula* and *Castanopsis*) accompanied by patchy coniferous trees (mainly *Pinus* and *Abies*) on the lower slopes. This pollen assemblage reflects a relatively warm and wet climate towards the beginning of the stage and slightly warmer and drier conditions later in the stage, which is compatible with the gradual increase seen in *Artemisia* and decrease in Polygonaceae.

The period between 22 and 13.9 cal. ka BP (Pollen Zone II) was marked by a large expansion of needle- and broad-leaved mixed forest (dominated by *Pinus*, *Abies* and *Quercus*), and a slight contraction of the steppe/grassland marked by notable reductions in Polygonaceae, *Artemisia* and Poaceae, coupled with increases in Cyperaceae and Asteraceae. Synchronously, the abundance of pteridophytes, particularly Polypodiaceae and Athyriaceae, decreased. This pollen assemblage suggests a warm and dry climate early in the stage followed by cold and wet conditions later in the stage.

Between 13.9 and 3 cal. ka BP (Pollen Zone III), the steppe/grassland once again expanded whilst cover of needle- and broad-leaved mixed forest reduced. Among the herbaceous plants, we noted a distinct rise in Polygonaceae accompanied by steady declines in *Artemisia*, Cyperaceae and Asteraceae. Pollen of the coniferous trees *Pinus* and *Abies*, and broad-leaved *Quercus* decreased markedly. *Betula* reached a peak in this period and pteridophytes increased compared with last phase. This pollen assemblage implies a fluctuating climate dominated by warm and humid conditions, with a marked cold and dry episode at the beginning of the phase, inferred from the abrupt rise of cold and drought tolerant *Betula* alongside a remarkable decline in *Quercus* and the low levels of Polygonaceae pollen.

From 3 cal. ka BP to the present day (Pollen Zone IV), the vegetation was characterized by an increase in needle-leaved forest and reduction of steppe/grassland. *Pinus* was the main coniferous pollen contributor, with a peak value of 35.5% for the entire core. *Abies* and *Quercus* retained low percentages. *Betula* almost vanished in this period. The herbs *Artemisia*, Polygonaceae, Cyperaceae and Poaceae together with the pteridophytes declined. This pollen assemblage suggests a relatively warm and dry climate in this period.

### Comparison with previous studies at Shudu Lake

Previously, Cook *et al*. [15, 16] conducted a multi-proxy analysis for a 635 cm-long core (06SD, Fig 1D) in order to understand Late Quaternary Asian monsoon variability at Shudu Lake. Jones *et al*. [13] investigated Holocene environmental change at this lake based on a 120 cm-long core (05SD1, Fig 1D). The pollen record of Cook *et al*. [15, 16] revealed that the vegetation in the Shudu Lake catchment was dominated by sparse xerophytic grassland/tundra or cold-tolerant boreal *Pinus* forest from ca. 22.6 to 17.7 ka BP, and shifted to steppe-tundra and cold/cool mixed forest between 17.7 and 10.7 ka BP. During the period of 10.7 to 10.1 ka BP, steppe-tundra vegetation expanded. From 10.1 to 6.7 ka BP, temperate deciduous and warm mixed forest expanded. After 6.7 ka BP, the uppermost sediments were missing from the core. Based on the pollen data, Jones *et al*. [13] pointed out that terrestrial vegetation cover in the lake catchment increased from 9.9 to 0.9 ka BP, was relatively stable from 0.9 to 0.6 ka BP, and decreased since 0.6 ka BP. In contrast, the present core, which documents a longer period of vegetational and climatic change than the earlier 06SD and 05SD1 cores, suggests that, during the past 30 ka BP, the vegetation went through a series of distinct changes, viz., steppe/grassland and broad-leaved forest (30 to 22 ka BP), large expansion of needle- and broad-leaved mixed forest and slight decline in steppe/grassland (22 to13.9 ka BP), expansion of steppe/grassland and decline of needle- and broad-leaved mixed forest (13.9 to 3 ka BP), expansion of needle-leaved forest and decrease in steppe/grassland (3 ka BP to the present day).

There are at least two possible explanations for the differences between the changes in vegetation observed in the present study and the earlier works. Firstly, we consider depositional conditions. The present core was extracted from a higher energy zone near the lake bank compared to the 06SD and 05SD1 cores which were taken in deeper water. Generally, lower energy zones are favorable for pollen deposition. The 06SD core is dominated by organic gyttja and silty clay and the 05SD1 core is abundant in organic rich silty clay and silty clay, while the present core is characterized by fine silt, clay with gravel, and grit with fine gravel. It is notable that the deposition of particles of the present core is larger than in the 06SD and 05SD1 cores. Secondly, there are differences in the dominant palynomorphs recovered from the cores: although the representative pollen types are similar, their pollen abundances are quite different. For example, *Pinus* is a dominant taxon of arboreal pollen. Its pollen abundance varies from c. 20–80% in the 06SD and 05SD1 cores, but is between zero and 35.5% in the present core. *Abies*, *Quercus* and *Betula* pollen also dominate in the three cores, yet occur at a variable frequency in each core. Of the non-arboreal pollen, Poaceae is prominent in the 06SD and 05SD1 cores with a peak of c. 40%, but Polygonaceae dominates in the present core with a maximum value of c. 80%. The differences observed between the three cores emphasize the value of having more than one study in the same place.

### Comparison with other sites in northwestern Yunnan

During the last two decades, a number of palynological investigations have been conducted on the Late Pleistocene and Holocene vegetational and climatic changes in the Hengduan Mountains of northwestern Yunnan [15, 16, 31–37]. If arranged into a descending altitudinal gradient, the study sites comprise Tiancai Lake (3898 m, [35]), Shudu Lake (3620 m, [15,16]), Haligu Lake (3277 m, [36]), the YXL profile (3310 m, [31]), Wenhai Lake (3080 m, [33]), Heqing Basin (2200 m, [32]), Xihu (1980 m, [34]) and Erhai Lake (1974 m, [37]) (Fig 1B). Herein we make a synthesis of all the published palynological literature on northwestern Yunnan to date, in order to build up a more complete understanding of the vegetational and climatic variability of the area.

Between 30 and 22 ka BP, the pollen assemblage at Shudu Lake was dominated by arboreal *Quercus* and *Betula* and herbaceous *Artemisia* and Polygonaceae, reflecting a vegetation of broad-leaved forest and steppe/grassland and indicating a warm, wet climate followed by slightly warmer, drier conditions. This assemblage is generally congruent with the pollen record of the YXL profile, a locality in Zhongdian about 300 m lower than Shudu Lake. During the period of c. 31 to 24.8 ka BP, the pollen assemblage of the YXL profile was characterized by the dominance of herbaceous *Festuca ovina* L. and *Gymnotheca involucrate* Pei, and arboreal *Salix* and *Fokienia hodginsii* (Dunn) Henry et Thomas, suggesting the vegetation shifting from alpine meadow to needle- and broad-leaved mixed forest, back to alpine meadow [31] (Fig 6). However, at Heqing Basin, a site about 1400 m lower than Shudu Lake, the pollen assemblage indicates forest dominated by *Quercus semecarpifolia* Sm., *Tsuga chinensis* (Franch.) Pritz. and *Pinus* sp. from 30 to 22.5 ka BP, with an increase in *Q*. *semecarpifolia* and *T*. *chinensis* between 26.5 and 23.7 ka BP, and an increase in *Pinus* and a decrease in *Q*. *semecarpifolia* and *T*. *chinensis* between 23.7 and 22.5 ka BP. This assemblage reflects a climatic shift from wet to dry, in agreement with that inferred from the Shudu Lake core [32] (Fig 6).

During the period of 22 to 13.9 ka BP, the pollen assemblage at Shudu Lake was dominated by an expansion of needle- and broad-leaved mixed forest (mainly comprising *Pinus*, *Abies*, *Quercus* and *Betula*) and a decrease in steppe/grassland species (primarily *Artemisia* and Polygonaceae), hinting at warm, dry conditions followed by a colder, wetter ones. In the YXL profile, from 21 to 13.6 ka BP, the pollen assemblage was marked by a decrease in herbaceous plants (*G*. *involucrata*, *Acorus calamus* L.) and increase in coniferous plants (mainly *F*. *hodginsii*), reflecting the alpine meadow vegetation shifting to needle-leaved forest [31] (Fig 6). At Wenhai, about 500 m lower than Shudu Lake, the pollen assemblage suggests that broad-leaved forest (dominated by *Quercus*, *Betula* and *Castanopsis*) gradually succeeded to needle-leaved forest (mainly composed of *Pinus* and *Abies*) between 22.5 and 9.25 ka BP [33] (Fig 6). At Heqing Basin, *Quercus* pollen content retained a high level and *Tsuga* began to decline between 22.5 and 14.2 ka BP, but aquatic pollen and pteridophytic spores increased greatly [32] (Fig 6). At Xihu and Eryuan, about 1600 m lower than Shudu Lake, the vegetation recorded was *Pinus*-evergreen sclerophyllous *Quercus* and montane conifers from 17 to 14 ka BP, pointing to a cold and semi-humid climate [34] (Fig 6).

From 13.9 to 3 ka BP, the pollen assemblage at Shudu Lake was marked by a decrease in arboreal pollen and prominent increase in Polygonaceae pollen and pteridophyte spores, indicating a decline in needle- and broad-leaved mixed forest and expansion of steppe/grassland. This assemblage points to a warm and humid climate entering the Holocene. In the period from 13.9 to 10.6 ka BP, cold- and drought-tolerant *Betula* reached a peak for the entire profile accompanied by low levels of *Quercus* pollen and a relatively high level of drought-tolerant Poaceae. We designate this short period as the Younger Dryas in the Shudu Lake core. At Tiancai Lake, about 280 m higher than Shudu Lake, the vegetation underwent a shift from alpine meadow to *Tsuga* forest between 12.2 and 2.9 ka BP [35] (Fig 6). At Haligu Lake, about 340 m lower than Shudu Lake, the vegetation underwent a shift from needle-leaved forest towards evergreen broad-leaved forest between 9.3 and 2.4 ka BP, again pointing to a warm and humid period. The evergreen *Quercus* phase (8.7–2.4 ka BP) was defined as the Holocene Optimum at Haligu core [36] (Fig 6). In the YXL profile, the pollen assemblage from 11.4 to 2.6 ka BP reflects a vegetation type of needle- and broad-leaved mixed forest dominated by *F*. *hodginsii*, *Juniperus formosana* Hayata., *Salix* and *Betula*, also suggesting warm and humid conditions. At Wenhai Lake, the vegetation was dominated by *Pinus-Abies*-*Tsuga* forest along with *Quercus-Betula* forest from 9.25 ka BP [33] (Fig 6). At Heqing Basin, the period from 14.2 to 3.7 ka BP was marked by frequent shifts in dominance between major pollen types, implying fluctuations between warm, dry and cold, wet conditions, with vegetation switching between needle- and broad-leaved forest and sparse woodland-steppe [32] (Fig 6). At Xihu, the vegetation between 14 and 10.5 ka BP was characterized by montane conifers, evergreen and deciduous forests, *Pinus* forests and semi-arid scrub, and after 10.5 ka BP by *Pinus* forests with evergreen broad-leaved components dominating all slopes [34] (Fig 6). At Erhai Lake, about 1600 m lower than Shudu Lake, evergreen broad-leaved *Quercus* forest and *Tsuga* forest went through a marked expansion between 12.9 and 6.4 ka BP, then declined from 6.4 to 2.2 ka BP accompanied by a notable increase in *Pinus* forest [37] (Fig 6).

After 3 ka BP, the pollen assemblage at Shudu Lake was characterized by a distinct increase in *Pinus* pollen, and a decrease in herbaceous pollen and pteridophyte spores, mirroring the expansion of needle-leaved forest and decline of steppe/grassland with a relatively warm and dry climate in this period. At Tiancai Lake, the vegetation was represented by the retreat of *Tsuga* forest since 2.9 ka BP, also indicating significant decreases in humidity [35] (Fig 6). At Haligu Lake, the vegetation was also a needle-leaved forest dominated by *Pinus* after 2.4 ka BP, but here increasing anthropogenic impact was involved during this period [36] (Fig 6). At Heqing Basin, *Pinus* forest dominated in the local vegetation from 3.7 ka BP to the present day [32] (Fig 6). However, at Erhai Lake, the vegetation was disturbed by human activities due to increased population immigration and the expansion of irrigation farming, evidenced by a rapid decline in *Pinus* pollen along with increases in Poaceae and fireweed pollen [37] (Fig 6).

In summary, regional comparisons reveal that the vegetation succession patterns in the Late Pleistocene and Holocene in northwestern Yunnan are certainly comparable, but clearly each site has its own characteristics due to the influence of altitude, topography, microclimate, etc., as well as human impacts. In general, however, we find that the kinds of transitions in vegetation occurring at different altitudes show a close correspondence to the altitudinal zonation present in modern day vegetation. Although some palynological investigations have low chronology and sampling resolutions, our synthesis provides a new insight into understanding the successional patterns of vegetation in northwestern Yunnan along an altitudinal gradient. In the future, more AMS ^14^C dates and pollen samples in this series of studies are needed to gather more detailed information on the Quaternary vegetational and climatic variation of this region. This may ultimately enable us to understand whether present day climate change is likely to see the characteristic vegetation zones we see today persisting at different altitudes or becoming less distinct and perhaps even replaced by novel ecosystems. The present evidence from this study, and the others considered here, suggests continuing coherence of the major vegetation zones.

## Supporting information

S1 Table Inferred ages based on age-depth model. (DOCX)
