# Supplementary material for: Pollen-based reconstruction of vegetational and climatic change over the past ~30 ka at Shudu Lake in the Hengduan Mountains of Yunnan, southwestern China
Source: PLoS One. 2017 Feb 9;12(2):e0171967. doi: 10.1371/journal.pone.0171967 (PMC5300144; doi:10.1371/journal.pone.0171967)
Supplement: S1 Table — (DOCX) [file pone.0171967.s001.docx]

**S1 Table. Inferred ages based on age-depth model**

| No. | Depth (cm) | Age (cal. ka BP) |
| --- | --- | --- |
| 1 | 10 | 0.7 |
| 2 | 20 | 1.5 |
| 3 | 30 | 2.2 |
| 4 | 40 | 3.0 |
| 5 | 50 | 3.8 |
| 6 | 60 | 4.6 |
| 7 | 71 | 5.4 |
| 8 | 80 | 6.9 |
| 9 | 90 | 7.9 |
| 10 | 100 | 8.9 |
| 11 | 110 | 9.9 |
| 12 | 120 | 10.9 |
| 13 | 130 | 11.9 |
| 14 | 140 | 12.9 |
| 15 | 150 | 13.9 |
| 16 | 160 | 14.9 |
| 17 | 170 | 15.9 |
| 18 | 181 | 17.0 |
| 19 | 190 | 17.9 |
| 20 | 200 | 18.9 |
| 21 | 210 | 19.9 |
| 22 | 220 | 20.9 |
| 23 | 230 | 21.9 |
| 24 | 240 | 22.9 |
| 25 | 250 | 23.9 |
| 26 | 260 | 24.9 |
| 27 | 270 | 25.9 |
| 28 | 280 | 26.9 |
| 29 | 290 | 27.9 |
| 30 | 300 | 28.9 |
| 31 | 310 | 29.9 |
